# Probiotics improve renal function, glucose, lipids, inflammation and oxidative stress in diabetic kidney disease: a systematic review and meta-analysis

**DOI:** 10.1080/0886022X.2022.2079522

**Published:** 2022-05-24

**Authors:** Yali Dai, Jingjing Quan, Lianlian Xiong, Yanfang Luo, Bin Yi

**Affiliations:** a Department of Nephrology, Third Xiangya Hospital, Central South University, Changsha, China; b Department of Nephrology, Yueyang People’s Hospital, Yueyang Hospital affiliated to Hunan Normal University, Yueyang, China

**Keywords:** Probiotics, diabetic kidney disease, renal function, glucose and lipid metabolism, inflammation, oxidative stress, meta-analysis

## Abstract

**Aims:**

The role of probiotics in the management of diabetic kidney disease (DKD) has been shown. Several current trials are investigating the effect of probiotics, which are widely used to modulate biomarkers of renal function, glucose, lipids, inflammation and oxidative stress in patients with DKD. However, their findings are controversial. This study aimed to systematically evaluate the impact of probiotics on patients with DKD *via* meta-analysis.

**Methods:**

PubMed, The Cochrane Library, Web of Science, Scopus, Embase, China National Knowledge Infrastructure, Chinese Wanfang Database and Chinese VIP Database were searched for relevant studies from the establishment of these databases to September 2021. The pooled results evaluated the impact of probiotics on renal function, glucose, lipids, inflammation and oxidative stress indicators in patients with DKD. Additionally, subgroup analysis was performed based on intervention duration, probiotic dose and probiotic consumption patterns, respectively.

**Results:**

Ten trials that included 552 participants were identified for analysis. Compared with the controls, probiotics significantly decreased serum creatinine (Scr) [WMD = −0.17 mg/dL; 95%CI = −0.29, −0.05; *p* = 0.004], blood urea nitrogen (BUN) [WMD = −1.36 mg/dL; 95%CI = −2.20, −0.52; *p* = 0.001], cystatin C (Cys C) [WMD = −29.50 ng/mL; 95%CI = −32.82, −26.18; *p* < 0.00001], urinary albumin/creatinine ratio (UACR) [WMD = −16.05 mg/g; 95%CI = −27.12, −4.99; *p* = 0.004] and natrium (Na) [WMD = −0.94 mmol/L; 95%CI = −1.82, −0.05; *p* = 0.04] in patients with DKD. Enhanced glycemic control was observed in patients with DKD receiving probiotics compared with controls, as demonstrated by reduced levels of fasting plasma glucose (FPG), hemoglobin A1c (HbA1c), homeostasis model of assessment-estimated insulin resistance (HOMA-IR), and increased quantitative insulin sensitivity check index (QUICKI). Probiotics affected lipid metabolism parameters with decreasing triglycerides (TG), total cholesterol (TC) and low-density lipoprotein cholesterol (LDL-c) levels in patients with DKD. Probiotics could also could improve inflammation and oxidative stress by decreasing high-sensitivity C-reactive protein (hs-CRP), plasma malondialdehyde (MDA), total antioxidant capacity (TAC), glutathione (GSH) and nitric oxide (NO). Additionally, subgroup analysis showed that those who received multiple species probiotics had a statistically significant difference in BUN, FPG, HOMA-IR, high-density lipoprotein cholesterol (HDL-c), MDA, TAC, and NO. Meanwhile, Scr, LDL-c, HDL-c, MDA, and TAC were ameliorated when the intervention duration was more than eight weeks and BUN, FPG, HOMA-IR, and MDA were improved when the probiotic dose was greater than four billion CFU/day.

**Conclusions:**

Our analysis revealed that probiotics could delay the progression of renal function injury, improve glucose and lipid metabolism, and reduce inflammation and oxidative stress in patients with DKD. Subgroup analysis showed that intervention duration, probiotic dose and probiotic consumption patterns had an effect of probiotics on outcomes.

Diabetic kidney disease (DKD), one of the most frequent complications of diabetes, is the major cause of end-stage kidney disease. Abnormal glucose and lipid metabolism, renal hemodynamic changes, oxidative stress, and immune-inflammatory responses are vital mechanisms for DKD [1,2]. A recent study reported [3] that 20–40% of diabetic patients suffer from DKD, and the global prevalence of DKD is 15.48 ‰ in males and 16.50 ‰ in females. In the United States, the mean annualized direct medical cost of patients with DKD is $6826 [4]. Based on the severe health impact and financial burden associated with DKD, it is particularly important to find effective interventions to mitigate DKD progression. Therefore, it is urgent to explore low-cost and effective treatment programs to reduce the morbidity and mortality of patients with DKD.

Probiotics are active microorganisms that are beneficial to the host [5]. *Lactobacillus (L) spp.*, *Bifidobacterium (B) spp.*, *Streptococcus spp.*, *Enterococcus spp.*, and *Saccharomyces boulardii* are the most commonly administered strains for supplementation [6]. By maintaining intestinal epithelial barrier function, competing with pathogens for nutrients and regulating the host immune response, probiotics can improve host metabolism, relieve uremic toxicity, reduce pro-inflammatory factor levels, and delay the progression of renal function injury [7,8]. A systematic review of Vlachou in animal and clinical studies [9] found that probiotics on subjects with DKD had significant alterations in biomarkers of inflammation and renal function in other biomarkers such as fasting plasma glucose (FPG), homeostasis model of assessment-estimated insulin resistance (HOMA-IR), increased quantitative insulin sensitivity check index (QUICKI), insulin, triglycerides (TG), very low-density lipoprotein cholesterol (VLDL-c) and high-density lipoprotein cholesterol (HDL-c) levels. However, the meta of Moravejolahkami [10] and AbdelQadir [11] demonstrated that probiotics had no beneficial effect on patients with DKD regarding lipid profiles such as TG, total cholesterol (TC), VLDL-c, HDL-c levels, and oxidative stress such as nitric oxide (NO), glutathione (GSH). Tarrahi [12] showed that there was no statistically significant change in hemoglobin A1c (HbA1c), insulin, QUICKI, blood urea nitrogen (BUN), and glomerular filtration rate (GFR) in patients with DKD. These conflicting results prompted us to systematically analyze available randomized controlled trials (RCTs), gaining a deeper and more precise understanding of the effects of probiotics on biomarkers in renal function injury, glucose and lipid metabolism, inflammation and oxidative stress in patients with DKD. Furthermore, this meta-analysis through subgroup analysis investigated the influence of intervention duration, probiotic dose and probiotic consumption patterns on included research biomarkers.

## 1. Materials and methods

### 1.1. Inclusion and exclusion criteria

Studies were selected for inclusion by two independent reviewers (YL.D and JJ.Q), and were approved by a third reviewer (LL.X). Inclusion criteria: (1) RCTs design; (2) DKD patients without restriction on age or medical conditions; (3) intervention using probiotics; (4) assessment of renal function injury, glucose and lipid metabolism, inflammation and oxidative stress mediator concentrations as an outcome variable. Exclusion criteria: (1) Non-RCTs design, such as observational studies, reviews, meta-analyses, short reports, conference papers, research projects, or animal trials. (2) RCTs with improper statistical methods, incomplete data, and undescribed data with mean and standard deviation; (3) Literature of poor quality or without full text.

### 1.2. Search strategy and selection studies

We implemented and reported the current study according to the preferred reporting items for systematic reviews and meta-analysis guideline (PRISMA). The systematic literature search (PubMed, The Cochrane Library, Web of Science, Scopus, Embase, China National Knowledge Infrastructure, Chinese Wanfang database and Chinese VIP database) was performed to identify literature published from the establishment of these databases to September 2021. We used MeSH terms and the following query: #1: (probiotics) OR (probiotic), #2: ((((diabetic nephropathy) OR (diabetic kidney disease)) OR (DKD)) OR (diabetic nephropathies)) OR (diabetic patients with nephropathy), #3: (#1) AND (#2). Additionally, a hand search was conducted among the citation lists of all known relevant publications and review studies to identify RCTs that were not captured by the online electronic searches. The above work was completed by the YL.D, JJ.Q. and YF.L.

### 1.3. Literature screenings and data extraction

After meeting the inclusion and exclusion criteria, the included studies were independently reviewed by two reviewers (YL.D and JJ.Q) using a standardized template independently. Agreement for inclusion was calculated using the Cohen *κ* [13]. Any discrepancies were discussed and resolved with a third reviewer (YF.L). The subsequent data were abstracted: (1) basic features: author, year, country, age, target population, intervention, study duration, and outcome biomarkers; (2) methods: randomization, allocation concealment, blindness, data integrity, selective reporting, and other biases; (3) research objects: patients with DKD were divided into an experimental group and a control group; (4) intervention measures: specific medication, dose, treatment duration; (5) outcome biomarkers: renal function: serum creatinine (Scr), BUN, GFR, 24-h urine protein (24 h-UP), urinary albumin/creatinine ratio (UACR), cystatin C (Cys C), potassium (K), natrium (Na); glucose metabolism: FPG, 2 h postprandial blood glucose (2 h-PBG), insulin, HbA1c, HOMA-IR, QUICKI; lipid metabolism: TG, TC, low-density lipoprotein cholesterol (LDL-c), VLDL-c, HDL-c; inflammation and oxidative stress: serum high-sensitivity C-reactive protein (hs-CRP), plasma malondialdehyde (MDA), total antioxidant capacity (TAC), GSH and NO.

### 1.4. Literature quality evaluations

The researchers (YL.D, JJ.Q. and YF.L.) evaluated the quality of the literature independently. The Cochrane bias risk assessment tool was used to assess methodological quality. Evaluation aspects included whether: (1) random sequences were properly generated; (2) the distribution of hidden was properly used; (3) subjects and intervention providers were properly blinded; (4) evaluators of the results were properly blinded; (5) the completeness of outcome data was properly maintained; (6) selective reporting was properly conducted; (7) other biases were properly disposed. According to the above specific evaluation criteria, the included studies were categorized as ‘low risk’, ‘high risk’ or ‘unclear risk’.

### 1.5. Statistical data analysis

Stata software version 16.0 and RevMan 5.3 were used for statistical analysis and graph of risk of bias. Cohen *κ* was calculated by SPSS 25.0. The effect of probiotics on selected parameters was analyzed using the mean difference with standard deviations (SDs). The weighted mean difference (WMD) was adopted when the same measurement unit or method was applied for the same intervention. Otherwise, the standardized mean difference (SMD) was employed. When the study’s authors did not provide SDs of mean differences, SDs for the changes from baseline were substituted using a correlation coefficient calculated according to Cochrane recommendations and the SDs for the baseline and final means for each group. Therefore, we calculated the SDs of outcomes using the following formula: SD^2^ change = SD^2^ baseline + SD^2^ final – (2 × correlation coefficient × SD baseline × SD final), assuming that the correlation coefficient I was 0.5 [14]. Interval estimation adopted a 95% confidence interval (CI). *I*^2^ ≤ 50% and *p* ≥ 0.05 implied a lack of heterogeneity; therefore, the fixed-effect model was used to combine the effect value. *I*^2^ > 50% and *p* < 0.05 indicated the existence of heterogeneity; hence, the random-effect model was used. Furthermore, potential publication bias in each analysis was assessed quantitatively using Egger’s test [15]. If publication bias existed, the results were reported truthfully after considering the sensitivity analysis results [16]. Sensitivity analysis was performed based on the characteristics of the study. A *p* value of 0.05 was considered as level of statistical significance.

## 2. Results

### 2.1. Literature retrieval, research baseline characteristics, and methodological quality assessment

**Figure 1.**
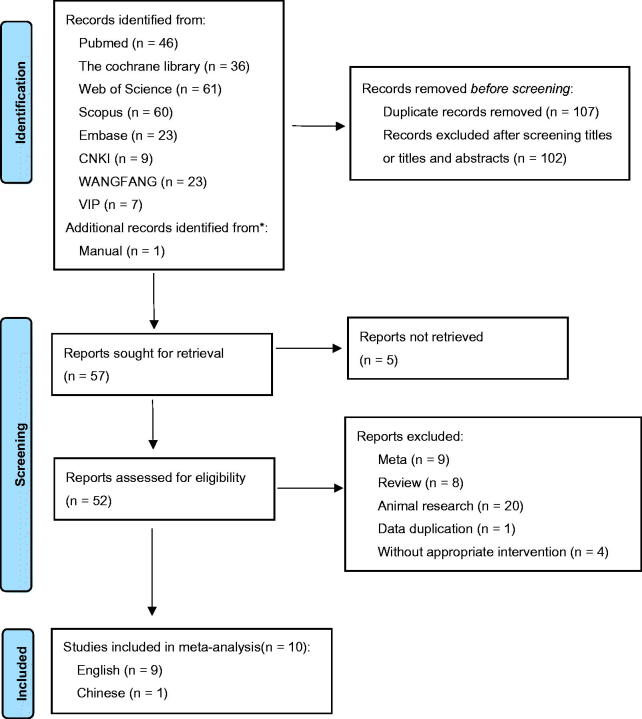
PRISMA flow diagram of the study selection process.

**Figure 2. Quality assessments of the included RCTs articles. (A) Risk of bias graph; (B) risk of bias summary for all RCT studies.**
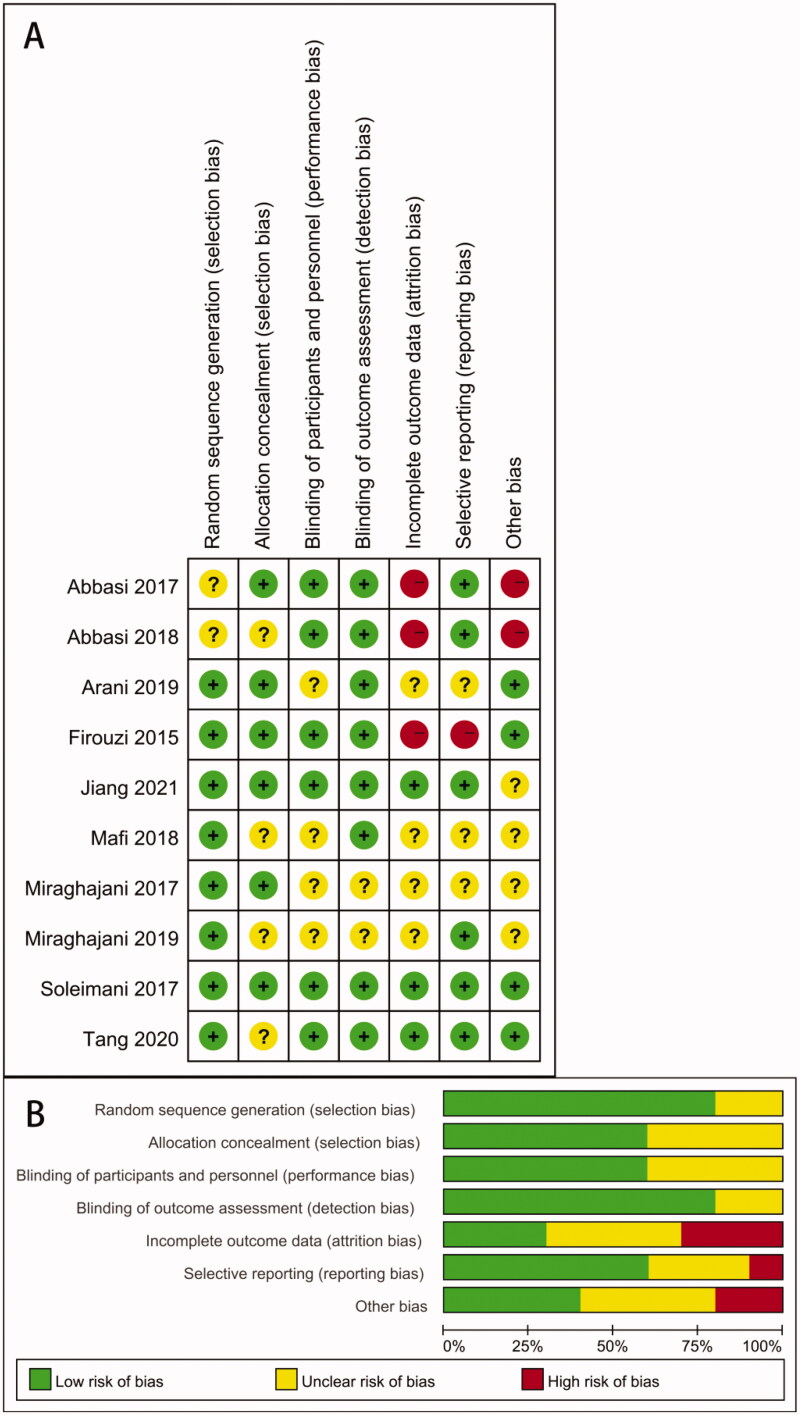
.

A total of 266 articles were searched through literature retrieval. After carefully reviewing the titles, abstracts, duplications and relevance, we retained 57 articles for further review. Interrater agreement in determining the final studies from the 57 screened citations was substantial (*κ* = 0.837). 5 reports without retrieved text, 9 meta-analyses, 8 reviews, 20 animal experiments, 1 study data duplication and 4 articles without appropriate intervention were further excluded. In the end, 10 RCTs were included for meta-analysis, containing 9 English articles and 1 Chinese article. The 10 RCTs incorporated a total of 552 participants (intervention, 280; control, 272) (Figure 1). The characteristics of all included RCTs were summarized in Table 1, with their methodological quality highlighted in Figure 2.

**Table 1.** Characteristics of the included randomized controlled trials in systematic review.

Author and year	Country	Participants (E/C)	Age(years) (E/C)	Target population	Intervention	Duration (weeks)	Outcome indexes
Firouzi 2015 [13]	Malaysia	136(68/68）	E:52.9 ± 9.2 C:54.2 ± 8.3	Diabetic kidney disease	Microbial cell preparation (*Lactobacillus acidophilus, Lactobacillus casei, Lactobacillus lactis, Bifidobacterium bifidum, Bifidobacterium longum and Bifidobacterium infantis*); Totally 6*10^10^ CFU/d	6 and 12	Scr, BUN, GFR, K, Na
Abbasi 2017 [17]	Iran	40 (20/20)	E:56.9 ± 8.1 C:53.6 ± 7.19	Diabetic kidney disease	Probiotic soy milk (*Lactobacillus plantarum A7*); Totally 4*10^9^ CFU/d	8	Scr, GFR, UACR
Miraghajani 2017 [18]	Iran	40 (20/20)	E:56.90 ± 1.81 C:53.60 ± 1.60	Diabetic kidney disease	Probiotic soy milk *(Lactobacillus plantarum A7)*; Totally 4*10^9^ CFU/d	8	TC MDA,TAC,GSH
Soleimani 2017 [7]	Iran	60 (30/30)	E:54.0 ± 16.0 C:59.4 ± 16.0	Diabetic Hemodialysis	Probiotic capsule (*Bifidobacterium bifidum, Lactobacilluscasei, and Lactobacillus acidophilus*); Totally6*10^9^ CFU/d	12	Scr, BUN, GFR, K, Na FPG, Insulin, HOMA-IR, QUICKI, HbA1c TG, TC, LDL-c, VLDL-c, HDL-c MDA, TAC, GSH, NO, hs-CRP
Abbasi 2018 [19]	Iran	40 (20/20)	E:56.9 ± 8.1 C:53.6 ± 7.19	Diabetic kidney disease	Probiotic soy milk (*Lactobacillus plantarum A7*); Totally 4*10^9^ CFU/d	8	Scr, GFR, TG, TC, LDL-c, HDL-c
Mafi 2018 [15]	Iran	60 (30/30)	E:58.9 ± 8.8 C:60.9 ± 4.4	Diabetic kidney disease	Probiotic supplements (*Lactobacillus acidophilus strain ZT-L1, Bifidobacterium bifidum strain ZT-B1, Lactobacillus Reuteri strain ZT-Lre, and Lactobacillus fermentum strain ZT-L3*); Totally 8*10^9^ CFU/d	12	Scr, BUN, GFR, 24 h-UP FPG, Insulin, HOMA-IR, QUICKI, HbA1c TG, TC, LDL-c, VLDL-c, HDL-c MDA, TAC, GSH, NO, hs-CRP
Arani 2019 [16]	Iran	60 (30/30)	E:62.7 ± 9.1 C:60.3 ± 8.5	Diabetic kidney disease	Probiotic honey (*Bacillus coagulans T11*) Totally 2.5*10^9^ CFU/d	12	Scr, BUN FPG, Insulin, HOMA-IR, QUICKI, TG, TC, LDL-c, VLDL-c, HDL-c MDA, TAC, GSH, NO, hs-CRP
Miraghajani 2019 [20]	Iran	40 (20/20)	E:56.90 ± 1.81 C:53.60 ± 1.60	Diabetic kidney disease	Probiotic soy milk (*Lactobacillus plantarum A7*); Totally 4*10^9^ CFU/d	8	Cys C, K
Tang 2020 [21]	China	90(45/45)	E:55.82 ± 6.36 C:56.86 ± 6.41	Diabetic kidney disease	Probiotic tablet (*Bifidobacterium, lactobacillus bulgaricus, and Live streptococcus thermophilus*); Totally 4.8*10^7^ CFU/d	12	Scr, BUN, Cys C, 24 h-UP FPG, HbA1c, 2 h-PBG LDL-c, HDL-c MDA, TAC, GSH, NO,
Jiang 2021 [22]	China	76(42/34)	E:55.96 ± 8.45 C:56.12 ± 8.23	Diabetic kidney disease	Probiotic supplements (*Bifidobacterium bifidum, Lactobacillus acidophilus, Streptococcus thermophilus*) Totally 3.2*10^9^ CFU/d	12	GFR, UACR FPG, HbA1c, 2 h-PBG

Abbreviations: E: intervention; C: control; Scr: serum creatinine; BUN: blood urea nitrogen; GFR: glomerular filtration rate; 24 h-UP: 24-h urine protein; UACR: urinary albumin/creatinine ratio; Cys C: cystatin C; K: potassium; Na: natrium; FPG: fasting plasma glucose; 2 h-PBG: 2 h postprandial blood glucose; HbA1c: hemoglobin A1c; HOMA-IR: homeostasis model of assessment-estimated insulin resistance; QUICKI: quantitative insulin sensitivity check index; TG: triglycerides; TC: total cholesterol; LDL-c: low-density lipoprotein cholesterol; VLDL-c: very low-density lipoprotein cholesterol; HDL-c: high-density lipoprotein cholesterol; hs-CRP: serum high-sensitivity C-reactive protein; MDA: malondialdehyde; TAC: total antioxidant capacity; GSH: glutathione; NO: nitric oxide.

### 2.2. Effects of probiotics on renal function

#### 2.2.1. Serum creatinine

**Figure 3.**
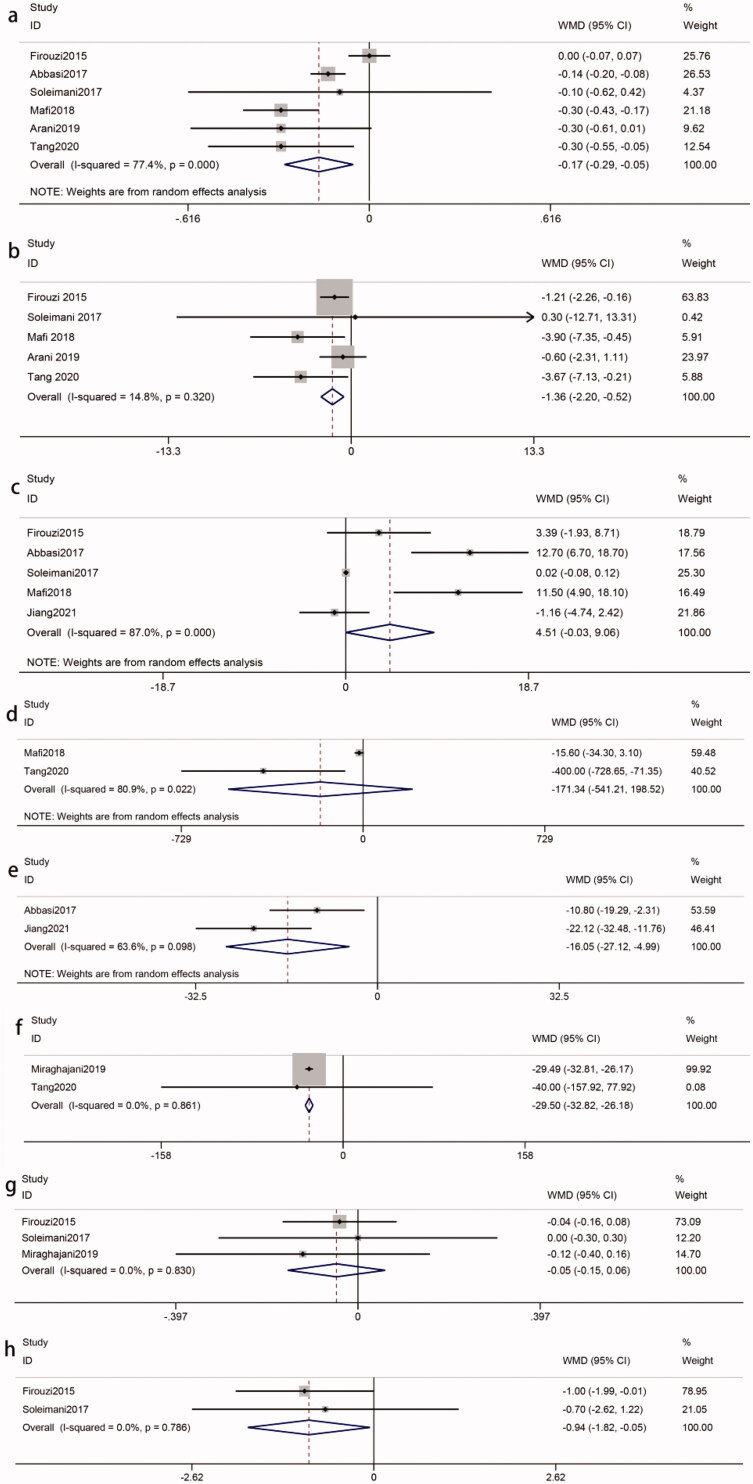
Effects of probiotics on biomarkers of renal function. (a) Scr. (b) BUN. (c) GFR. (d) 24 h-UP (e) UACR (f) Cys c (g) K (h) Na. Abbreviations refer to Table 1.

The efficacy of probiotics on Scr was reported by six studies [8,17,19–22] with 446 participants (intervention, 223; control, 223). A significant reduction was observed in patients who received treatment (WMD = −0.17 mg/dL; 95% CI = −0.29, −0.05; *p* = 0.004) with heterogeneity (*I*^2^ = 77%, *p* = 0.0005) (Figure 3(a)). Sensitivity analysis identified that the Firouzi et al. study [21] contributed to the heterogeneity. After excluding the study, the heterogeneity had an improvement (*I*^2^ = 40%, *p* = 0.16). The reduction in Scr was still significant (WMD = −0.22 mg/dL; *p* < 0.0001). Additionally, we also conducted a subgroup analysis to find possible sources. Any subgroup could not explain the between-study heterogeneity. Interestingly, the subsets of ‘intervention duration > 8 weeks’ (WMD = −0.20 mg/dL; *p* = 0.04) and ‘probiotic doses ≤ 4*10**^9^** CFU/d’ (WMD = −0.18 mg/dL; *p* = 0.0003) were greater than the overall results (Table 2).

**Table 2.** Subgroup analysis of biomarkers.

Biomarkers	Subgroup	*n*	WMD or SMD (95%CI)	*I*^2^(%)	^a^ *p*	^b^ *p*	^c^ *p*
Scr	Total	6	−0.17 (−0.29, −0.05)	77	0.004	0.0005	
	Intervention duratio*n* ≤ 8 weeks	1	−0.14 (−0.20, −0.08)	–	<0.00001	–	0.58
	Intervention duratio*n* > 8 weeks	5	−0.20 (−0.39, −0.01)	81	0.04	0.0003	
	Single species	2	−0.15 (−0.20, −0.09)	0	<0.00001	0.32	0.79
	Multiple species	4	−0.18 (−0.39,0.04)	84	0.11	0.0003	
	Dos*e* ≤ 4*10^9^ CFU/d	3	−0.18 (−0.27, −0.08)	17	0.0003	0.30	0.78
	Dos*e* > 4*10^9^ CFU/d	3	−0.14 (−0.39, 0.12)	87	0.29	0.0003	
BUN	Total	5	−1.36 (−2.20, −0.52)	15	0.001	0.32	
	Single species	1	−0.60 (−2.31, 1.11)	–	0.49	–	0.32
	Multiple species	4	−1.60 (−2.56, −0.64)	19	0.001	0.30	
	Dos*e* ≤ 4*10^9^ CFU/d	2	−1.21 (−2.74, 0.33)	59	0.12	0.12	0.81
	Dos*e* > 4*10^9^ CFU/d	3	−1.43 (−2.43, −0.43)	9	0.005	0.33	
GFR	Total	5	4.51 (−0.03, 9.06)	87	0.05	<0.00001	
	Intervention duratio*n* ≤ 8 weeks	1	12.70 (6.70, 18.70)	–	<0.0001	–	0.004
	Intervention duratio*n* > 8 weeks	4	2.33 (−1.39, 6.05)	78	0.22	0.004	
	Single species	1	12.70 (6.70, 18.70)	–	<0.0001	–	0.004
	Multiple species	4	2.33 (−1.39, 6.05)	78	0.22	0.004	
	Dos*e* ≤ 4*10^9^ CFU/d	2	5.55 (−8.02, 19.13)	93	0.42	0.0001	0.87
	Dos*e* > 4*10^9^ CFU/d	3	4.29 (−1.95, 10.52)	85	0.18	0.001	
K	Total	3	−0.05 (−0.15, 0.06)	0	0.39	0.83	
	Intervention duratio*n* ≤ 8 weeks	1	−0.12 (−0.40, 0.16)	–	0.40	–	0.57
	Intervention duratio*n* > 8 weeks	2	−0.03 (−0.15, 0.08)	0	0.56	0.81	
	Single species	1	−0.12 (−0.40, 0.16)	–	0.40	–	0.57
	Multiple species	2	−0.03 (−0.15, 0.08)	0	0.56	0.81	
	Dos*e* ≤ 4*10^9^ CFU/d	1	−0.12 (−0.40, 0.16)	–	0.40	–	0.57
	Dos*e* > 4*10^9^ CFU/d	2	−0.03 (−0.15, 0.08)	0	0.56	0.81	
FPG	Total	5	−13.53 (−19.85, −7.21)	48	<0.0001	0.10	
	Single species	1	−7.30 (−17.33, 2.73)	–	0.15	–	0.12
	Multiple species	4	−17.63 (−25.78, −9.49)	42	<0.0001	0.16	
	Dos*e* ≤ 4*10^9^ CFU/d	3	−9.87 (−17.03, −2.72)	34	0.007	0.22	0.03
	Dos*e* > 4*10^9^ CFU/d	2	−26.50 (−39.98, −13.02)	0	0.0001	0.78	
Insulin	Total	3	−3.87 (−7.51, −0.22)	89	0.04	0.0001	
	Single species	1	−1.10 (−1.89, −0.31)	–	0.007	–	0.12
	Multiple species	2	−5.64 (−11.32, 0.03)	86	0.05	0.007	
	Dos*e* ≤ 4*10^9^ CFU/d	1	−1.10 (−1.89, −0.31)	–	0.007	–	0.12
	Dos*e* > 4*10^9^ CFU/d	2	−5.64 (−11.32, 0.03)	86	0.05	0.007	
HbA1c	Total	4	−0.12 (−0.20, −0.04)	28	0.002	0.24	
	Dos*e* ≤ 4*10^9^ CFU/d	2	−0.47 (−0.90, −0.04)	0	0.03	0.83	0.10
	Dos*e* > 4*10^9^ CFU/d	2	−0.11 (−0.19, −0.03)	31	0.006	0.23	
HOMA-IR	Total	3	−1.99 (−3.99, 0.01)	91	0.05	<0.0001	
	Single species	1	−0.50 (−0.76, −0.24)	–	0.0001	–	0.02
	Multiple species	2	−2.87 (−4.83, −0.91)	73	0.004	0.06	
	Dos*e* ≤ 4*10^9^ CFU/d	1	−0.50 (−0.76, −0.24)	–	0.0001	–	0.02
	Dos*e* > 4*10^9^ CFU/d	2	−2.87 (−4.83, −0.91)	73	0.004	0.06	
QUICKI	Total	3	0.02 (0.00, 0.03)	93	0.01	<0.00001	
	Single species	1	0.01 (0.00, 0.01)	–	0.002	–	0.28
	Multiple species	2	0.03 (−0.01, 0.07)	96	0.18	<0.00001	
	Dos*e* ≤ 4*10^9^ CFU/d	1	0.01 (0.00, 0.01)	–	0.002	–	0.28
	Dos*e* > 4*10^9^ CFU/d	2	0.03 (−0.01, 0.07)	96	0.18	<0.00001	
TG	Total	4	−11.23 (−24.55, 2.10)	74	0.1	0.009	
	Intervention duratio*n* ≤ 8 weeks	1	−10.70 (−17.53, −3.87)	–	0.002	–	0.84
	Intervention duratio*n* > 8 weeks	3	−13.23 (−36.69, 10.23)	82	0.27	0.004	
	Single species	2	−8.73 (−15.54, −1.92)	12	0.01	0.29	0.62
	Multiple species	2	−20.60 (−66.43, 25.23)	90	0.38	0.001	
	Dos*e* ≤ 4*10^9^ CFU/d	2	−8.73 (−15.54, −1.92)	12	0.01	0.29	0.62
	Dos*e* > 4*10^9^ CFU/d	2	−20.60 (−66.43, 25.23)	90	0.38	0.001	
TC	Total	5	−6.93 (−11.67, −2.19)	0	0.004	0.66	
	Intervention duratio*n* ≤ 8 weeks	2	−7.50 (−14.05, −0.94)	0	0.03	0.97	0.81
	Intervention duratio*n* > 8 weeks	3	−6.32 (−13.16, 0.53)	15	0.07	0.31	
	Single species	3	−7.52 (−13.11, −1.94)	0	0.008	1.00	0.69
	Multiple species	2	−5.40 (−14.36, 3.55)	56	0.24	0.13	
	Dos*e* ≤ 4*10^9^ CFU/d	3	−7.52 (−13.11, −1.94)	0	0.008	1.00	0.69
	Dos*e* > 4*10^9^ CFU/d	2	−5.40 (−14.36, 3.55)	56	0.24	0.13	
LDL-c	Total	5	−7.14 (−11.03, −3.24)	0	0.0003	0.81	
	Intervention duratio*n* ≤ 8weeks	1	−7.00 (−12.10, −1.90)	–	0.007	–	0.94
	Intervention duratio*n* > 8weeks	4	−7.33 (−13.37, −1.29)	0	0.02	0.66	
	Single species	2	−7.29 (−11.87, −2.71)	0	0.002	0.80	0.90
	Multiple species	3	−6.73 (−14.15, 0.68)	0	0.08	0.47	
	Dos*e* ≤ 4*10^9^ CFU/d	3	−7.61 (−12.01, −3.20)	0	0.0007	0.85	0.65
	Dos*e* > 4*10^9^ CFU/d	2	−5.45 (−13.78, 2.89)	7	0.20	0.30	
VLDL-c	Total	3	−2.67 (−7.36, 2.01)	82	0.26	0.004	
	Single species	1	−0.60 (−3.23, 2.03)	–	0.66	–	0.47
	Multiple species	2	−4.12 (−13.33, 5.08)	90	0.38	0.001	
	Dos*e* ≤ 4*10^9^ CFU/d	1	−0.60 (−3.23, 2.03)	–	0.66	–	0.47
	Dos*e* > 4*10^9^ CFU/d	2	−4.12 (−13.33, 5.08)	90	0.38	0.001	
HDL-c	Total	5	2.71 (0.47, 4.97)	81	0.02	0.0003	
	Intervention duratio*n* ≤ 8 weeks	1	0.21 (−1.49, 1.91)	–	0.81	–	0.04
	Intervention duratio*n* > 8 weeks	4	3.49 (0.81, 6.17)	79	0.01	0.002	
	Single species	2	0.89 (−0.27, 2.04)	7	0.13	0.30	0.05
	Multiple species	3	4.41 (1.12, 7.69)	73	0.009	0.03	
	Dos*e* ≤ 4*10^9^ CFU/d	3	1.79 (−0.50, 4.08)	70	0.12	0.04	0.45
	Dos*e* > 4*10^9^ CFU/d	2	3.67 (−0.63, 7.98)	84	0.09	0.01	
hs-CRP	Total	3	−1.55 (−2.19, −0.91)	0	<0.00001	0.76	
	Single species	1	−1.70 (−2.99, −0.41)	–	0.01	–	0.79
	Multiple species	2	−1.50 (−2.23, −0.77)	0	<0.0001	0.49	
	Dos*e* ≤ 4*10^9^CFU/d	1	−1.70 (−2.99, −0.41)	–	0.01	–	0.52
	Dos*e* > 4*10^9^CFU/d	2	−2.76 (−5.68, 0.17)	87	0.06	0.006	
MDA	Total	5	−0.66 (−1.16, −0.16)	95	0.01	<0.00001	
	Intervention duratio*n* ≤ 8 weeks	1	0.01 (−0.07, 0.09)	–	0.79	–	<0.00001
	Intervention duratio*n* > 8 weeks	4	−0.77 (−0.94, −0.61)	0	<0.00001	0.48	
	Single species	2	−0.31 (−1.01, 0.38)	91	0.37	0.001	0.19
	Multiple species	3	−0.80 (−1.01, −0.59)	13	<0.00001	0.32	
	Dos*e* ≤ 4*10^9^ CFU/d	3	−0.51 (−1.18, 0.16)	95	0.13	<0.00001	0.43
	Dos*e* > 4*10^9^ CFU/d	2	−0.85 (−1.35, −0.34)	41	0.001	0.19	
TAC	Total	5	0.64 (0.41, 0.87)	49	<0.00001	0.10	
	Intervention duratio*n* ≤ 8 weeks	1	0.61 (−0.02, 1.25)	–	0.06	–	0.93
	Intervention duratio*n* > 8 weeks	4	0.64 (0.39, 0.89)	61	<0.00001	0.05	
	Single species	2	0.41 (0.02, 0.81)	0	0.04	0.43	0.18
	Multiple species	3	0.75 (0.47, 1.03)	63	<0.00001	0.07	
	Dos*e* ≤ 4*10^9^ CFU/d	3	0.74 (0.44, 1.04)	69	<0.00001	0.04	0.29
	Dos*e* > 4*10^9^ CFU/d	2	0.49 (0.12, 0.85)	0	0.009	0.64	
NO	Total	4	3.33 (−1.67, 8.33)	89	0.19	<0.00001	
	Single species	1	−0.50 (−1.45, 0.45)	–	0.30	–	0.03
	Multiple species	3	5.26 (0.25, 10.27)	69	0.04	0.04	
	Dos*e* ≤ 4*10^9^ CFU/d	2	4.17 (−5.32, 13.66)	96	0.39	<0.00001	0.82
	Dos*e* > 4*10^9^ CFU/d	2	2.98 (−0.12, 6.08)	0	0.06	0.66	
GSH	Total	4	72.74 (24.19, 121.28)	74	0.003	0.010	
	Intervention duratio*n* ≤ 8 weeks	1	111.3 (76.02, 146.58)	–	<0.00001	–	0.10
	Intervention duratio*n* > 8 weeks	3	56.52 (1.17, 111.87)	65	0.05	0.06	
	Single species	2	71.26 (−9.08, 151.60)	88	0.08	0.004	0.97
	Multiple species	2	73.23 (−8.51, 154.97)	68	0.08	0.08	
	Dos*e* ≤ 4*10^9^ CFU/d	2	71.26 (−9.08, 151.60)	88	0.08	0.004	0.97
	Dos*e* > 4*10^9^ CFU/d	2	73.23 (−8.51, 154.97)	68	0.08	0.08	

^a^*p* values for effect value; ^b^*p* values for heterogeneity within each subgroup; ^c^*p* values for between subgroup heterogeneity. (*p* < 0.05 indicate significant differences). Abbreviations refer to Table 1.

#### 2.2.2. Blood urea nitrogen

Five studies [8,17,19,21,22] included a total of 406 participants (intervention, 203; control, 203). There was a significant effect of probiotics on BUN (WMD = −1.36 mg/dL; 95%CI = −2.20, −0.52; *p* = 0.001) with no heterogeneity (*I*^2^ = 15%, *p* = 0.32) (Figure 3(b)). Subgroup analysis demonstrated that the impact of probiotics on BUN reduction toward the subsets of ‘multiple species probiotics’ (WMD = −1.60 mg/dL, *p* = 0.001) and ‘probiotic dose > 4*10^9^ CFU/d’ (WMD = −1.43 mg/dL, *p* = 0.005) was statistically significant and that was better than the overall results (Table 2).

#### 2.2.3. Glomerular filtration rate

Five RCTs [8,17,18,21,22], including 190 experimental participants and 182 controls, examined the effect of probiotics on GFR levels with heterogeneity between the studies (*I*^2^ = 87%, *p* < 0.00001). There was no statistical significance among the studies (WMD = 4.51 mL/min/1.73m^2^; 95%CI = −0.03, 9.06; *p* = 0.05) (Figure 3(c)). There was no significant difference after removing one single study using sensitivity analysis. Subgroup analysis was also performed to find the possible source of heterogeneity. However, any subgroup did not have a good explanation for the study heterogeneity (Table 2).

#### 2.2.4. 24-Hour urine protein

Two RCTs [17,19] investigated the impact of probiotics administration on 24 h-UP (subjects, 150; intervention, 75; control, 75). Overall, probiotics could not make a reduction in 24 h-UP levels (WMD = −171.34 mg/day; 95%CI = −541.21, 198.52; *p* = 0.36). The results were homogeneous (*I*^2^ = 81%, *p* = 0.02) (Figure 3(d)).

#### 2.2.5. Urinary albumin/creatinine ratio

There were two RCTs [18,22] that included a total of 116 participants (intervention, 62; control, 54). The pooled estimate demonstrated a significant reduction in UACR levels as a result of probiotic intervention (WMD = −16.05 mg/g; 95%CI = −27.12, −4.99; *p* = 0.004). Heterogeneity was recognized (*I*^2^ = 64%, *p* = 0.10) (Figure 3(e)).

#### 2.2.6. Cystatin C

The effect of probiotics on Cys-c was evaluated in two RCTs [19,23]. A total of 130 participants (subjects, 130; intervention, 65; control, 65) were involved. There was a significant impact on improving Cys C levels (WMD = −29.50 ng/mL; 95%CI = −32.82, −26.18; *p* < 0.00001) with no heterogeneity (*I*^2^ = 0%, *p* = 0.86) (Figure 3(f)).

#### 2.2.7. Potassium

Three RCTs [8,21,23] examined the effects of probiotics on K levels among 236 patients with DKD (intervention, 118; control, 118). The pooled results indicated that there was no significant decrease in the K levels compared to that in the control groups (WMD = −0.05 mmol/L; 95%CI = −0.15, 0.06; *p* = 0.39). The pooled results were not heterogeneous (*I*^2^ = 0%, *p* = 0.83) (Figure 3(g)). Subgroup analyses based on intervention duration, probiotic dose and probiotic consumption patterns were not statistically significant (*p* > 0.05) (Table 2).

#### 2.2.8. Natrium

Two RCTs [7,13] analyzed the effects of probiotics on Na levels with 196 participants (intervention, 98; control, 98). Data pooling showed that there was a significant reduction in Na levels following probiotic administration (WMD = −0.94 mmol/L; 95%CI = −1.82, −0.05; *p* = 0.04). Heterogeneity did not exist between studies (*I*^2^ = 0%, *p* = 0.79) (Figure 3(h)).

### 2.3. Effects of probiotics on glucose metabolism

#### 2.3.1. Fasting plasma glucose

**Figure 4.**
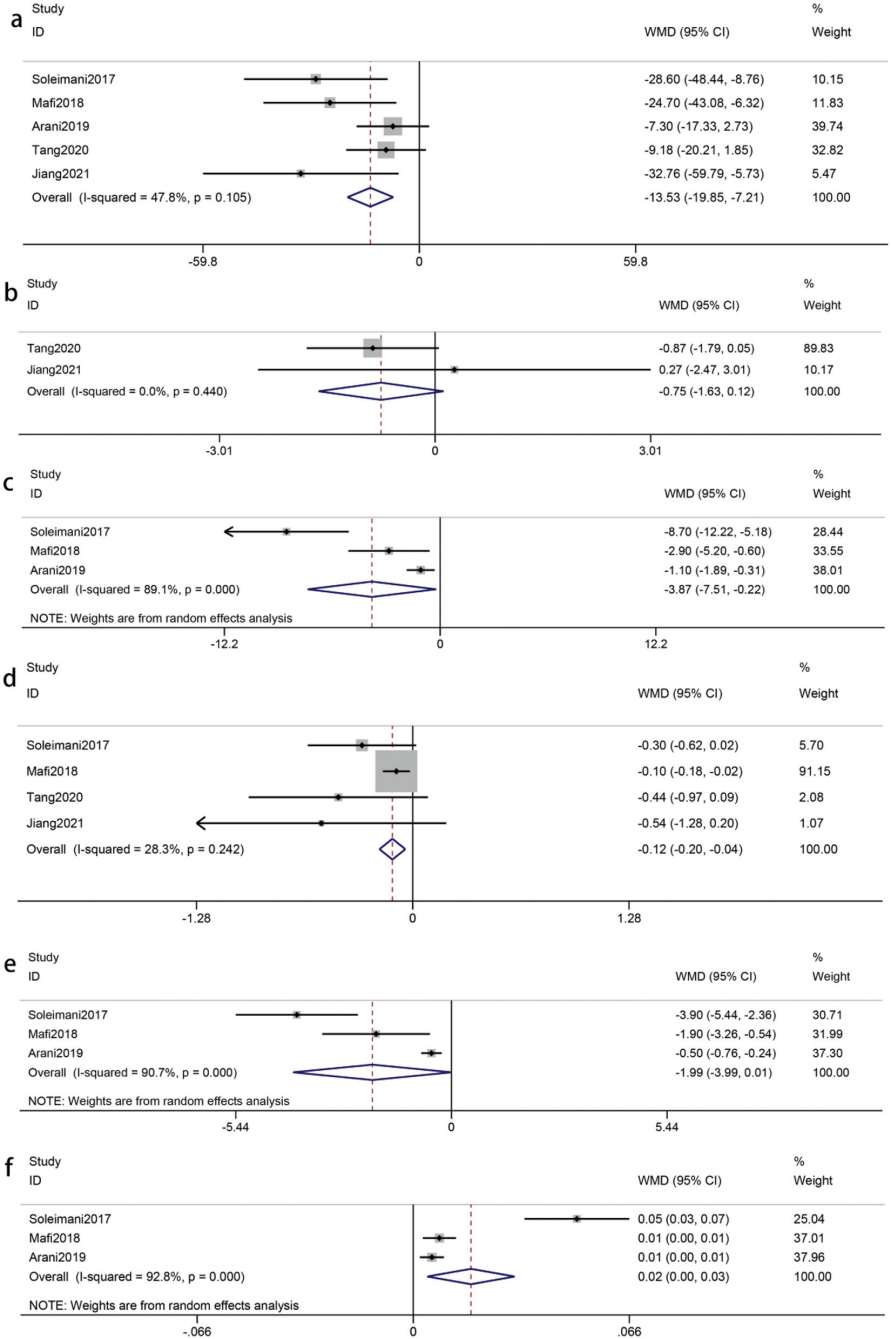
Effects of probiotics on biomarkers of glucose metabolism. (a) FPG. (b) 2h-PBG. (c) Insulin. (d) HbA1c. (e) HOMA-IR. (f) QUICKI. Abbreviations refer to Table 1.

Five studies [8,17–20], with 346 participants (intervention, 177; control, 169), reported the effects of probiotics on FPG. The results showed that the difference in FPG reduction between the probiotics-treated groups and control groups was significant (WMD = −13.53 mg/dL; 95%CI = −19.85, −7.21; *p* < 0.0001). No heterogeneity was recognized (*I*^2^ = 48%, *p* = 0.10) (Figure 4(a)). Further subgroup analysis demonstrated that the results based on multiple species probiotics (WMD = −17.63 mg/dL, *p* < 0.0001) and probiotic dose > 4*10**^9^** CFU/d (WMD = −26.50 mg/dL, *p* = 0.0001) were statistically significant (Table 2).

#### 2.3.2. 2 h postprandial blood glucose

Two studies [18,19] reported the effect of probiotics on 2 h-PBG among 166 participants (intervention, 87; control, 79) with no heterogeneity (*I*^2^ = 0%, *p* = 0.44). There was no significant decrease in the 2 h-PBG levels compared to controls (WMD = −0.75 mmol/L; 95%CI = −1.63, 0.12; *p* = 0.09) (Figure 4(b)).

#### 2.3.3. Insulin

The effects of probiotics on insulin were evaluated in 180 participants (intervention, 90; control, 90) from three studies [8,17,20]. There was heterogeneity between studies (*I*^2^ = 89%, *p* = 0.0001). The results demonstrate that the reduction in insulin was statistically significant (WMD = −3.87 uIU/mL, 95%CI = −7.51, −0.22; *p* = 0.04) (Figure 4(c)). By using sensitivity analysis, the Soleimani et al. study [8] contributed to the heterogeneity. Upon the exclusion, the heterogeneity was reduced (*I*^2^ = 53%, *p* = 0.15), but the effect of probiotics on insulin became nonsignificant (WMD = −1.66 uIU/mL; *p* = 0.05). The subgroup analysis did not have a good explanation for the source of heterogeneity (Table 2).

#### 2.3.4. Hemoglobin A1c

Four studies [8,17–19] with a total sample of 286 participants (intervention, 147; control, 139) measured the HbA1c between the intervention and control groups. The meta-analysis showed that there was a statistically significant difference in HbA1c (WMD = −0.12%, 95%CI = −0.20, −0.04; *p* = 0.002). There was evidence of no heterogeneity (*I*^2^ = 28%, *p* = 0.24) (Figure 4(d)). Interestingly, the subgroup analysis found that probiotic dose ≤ 4*10^9^ CFU/day had more apparent improvement (WMD = −0.47%, *p* = 0.03) (Table 2).

#### 2.3.5. Homeostasis model of assessment-estimated insulin resistance

Three RCTs [8,17,20], involving 180 participants (intervention, 90; control, 90), were included to evaluate the impact of probiotics on HOMA-IR. The results showed that there was no significant difference in HOMA-IR (WMD = −1.99, 95%CI = −3.99, 0.01; *p* = 0.05) with heterogeneity (*I*^2^ = 91%, *p* < 0.0001) (Figure 4(e)). However, after removing the Arani et al. study [20], the overall results turned statistically significant by using sensitivity analysis (WMD = −2.87; *p* = 0.004) and the heterogeneity was reduced (*I*^2^ = 73%, *p* = 0.06). Performing subgroup analysis showed that the effects of HOMA-IR in studies with multiple species probiotic and probiotic doses > 4*10^9^ CFU/day were more obvious (WMD = −2.87; *p* = 0.004) (Table 2).

#### 2.3.6. Quantitative insulin sensitivity check index

Three RCTs [8,17,20], involving 180 participants (intervention, 90; control, 90), reported QUICKI as the outcome biomarker. Our analysis suggested that probiotics had a significant impact on QUICKI (WMD = 0.02, 95%CI = 0.00, 0.03; *p* = 0.01). There was heterogeneity between these studies (*I*^2^ = 93%, *p* < 0.00001) (Figure 4(f)). A sensitivity analysis showed that the high heterogeneity was derived from the Soleimani et al. study [8]. The omission of the study reduced the heterogeneity (*I*^2^ = 0%, *p* = 0.47). And the reduction in QUICKI was still significant (WMD = 0.01; *p* < 0.0001). Subgroup analysis based on probiotic dose and probiotic consumption patterns did not reveal the source of heterogeneity (Table 2).

### 2.4. Effects of probiotics on lipid metabolism

#### 2.4.1. Triglycerides

**Figure 5.**
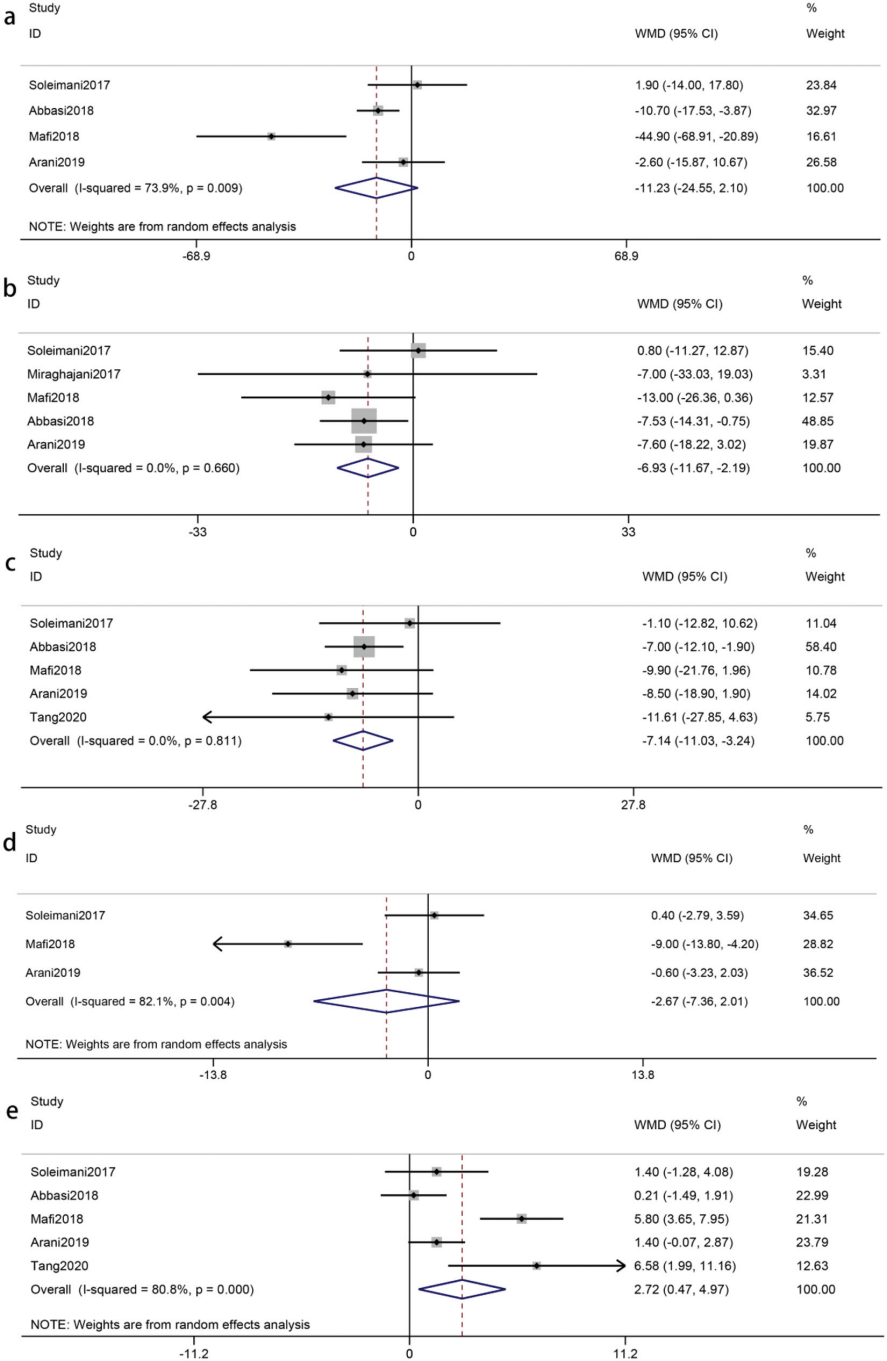
Effects of probiotics on biomarkers of lipid metabolism. (a) TG. (b) TC. (c) LDL-c. (d) VLDL-c. (e) HDL-c. Abbreviations refer to Table 1.

Four studies [8,17,20,24], involving 220 participants (intervention, 110; control, 110), reported the effects of probiotics on TG. Heterogeneity was recognized (*I*^2^ = 74%, *p* = 0.009). The meta-analysis of these studies revealed that the effect of probiotics on TG was not significantly reduced in patients with DKD (WMD = −11.23 mg/dL, 95%CI = −24.55, 2.10; *p* = 0.10) (Figure 5(a)). A sensitivity analysis showed that the heterogeneity was derived from the Mafi et al. study [17]. A heterogeneity became an acceptable level after removing it (*I*^2^ = 26%, *p* = 0.26). And a significant reduction in the TG could be observed (WMD = −6.45 mg/dL, *p* = 0.09). Subgroup analysis could not find the source of heterogeneity. And the intervention duration ≤ 8 weeks (WMD = −10.70 mg/dL, *p* = 0.002), probiotic dose ≤ 4*10^9^ CFU/day (WMD = −8.73 mg/dL, *p* = 0.01) and single-species probiotics (WMD = −8.73 mg/dL, *p* = 0.01) were statistically significant but were inferior to the overall results (Table 2).

#### 2.4.2. Total cholesterol

Five studies [8,17,20,24,25] reported TC levels from 260 participants (intervention, 130; control, 130). Heterogeneity between studies was not recognized (*I*^2^ = 0%, *p* = 0.66). Meta-analysis showed a significant reduction in TC in response to probiotics (WMD = −6.93 mg/dL, 95%CI = −11.67, −2.19; *p* = 0.004) (Figure 5(b)). Our study indicated that the decreased TC was more apparent in intervention duration ≤ 8 weeks (WMD = −7.50 mg/dL, *p* = 0.03), probiotic doses ≤ 4*10^9^ CFU/day (WMD = −7.52 mg/dL, *p* = 0.008) and single species probiotics (WMD = −7.52 mg/dL, *p* = 0.008). Interestingly, these results were better than the overall results (Table 2).

#### 2.4.3. Low-density lipoprotein cholesterol

Five studies [8,17,19,20,24] of 310 participants (intervention, 155; control, 155) were included to compare LDL-c levels in patients with DKD. Heterogeneity between those five studies was not recognized (*I*^2^ = 0%, *p* = 0.81). There was an obvious decline in the LDL-c levels in patients with DKD after probiotics (WMD = −7.14 mg/dL, 95%CI = −11.03, −3.24; *p* = 0.0003) (Figure 5(c)). Subgroup analysis suggested that probiotics was more significantly reduced LDL-c after an intervention duration > 8 weeks (WMD = −7.33 mg/dL, *p* = 0.02). However, probiotic doses ≤ 4*10^9^ CFU/day (WMD = −7.61 mg/dL, *p* = 0.0007) and single species probiotics (WMD = −7.29 mg/dL, *p* = 0.002) were better than the overall results (Table 2).

#### 2.4.4. Very low-density lipoprotein cholesterol

A meta-analysis evaluating the effect of probiotics on serum VLDL-c levels in patients with DKD was performed with data from three RCTs [8,17,20] (intervention, 90; control, 90). There was a significant between-study heterogeneity (*I*^2^ = 82%, *p* = 0.004). Our analysis showed that compared with the control, probiotics did not lead to a considerable decrease in serum VLDL-c levels in patients with DKD (WMD = −2.67 mg/dL, 95%CI = −7.36, 2.01; *p* = 0.26) (Figure 5(d)). After using sensitivity analysis, the overall effect was not significantly different, but the heterogeneity improved after removing the Mafi et al. study [17] (*I*^2^ = 0%, *p* = 0.64). Subgroup analysis suggested that no significant changes were observed in VLDL-C based on probiotic dose and probiotic consumption patterns (*p* > 0.05), which could not explain the source of heterogeneity (Table 2).

#### 2.4.5. High-density lipoprotein cholesterol

Data from a total of 310 participants (intervention, 155; control, 155) of five RCTs [8,17,19,20,24] were used to assess the impact of probiotics on HDL-c. Heterogeneity was found (*I*^2^ = 81%, *p* = 0.0003). Our study found that probiotics were positively associated with an increased level of serum HDL-c in patients with DKD (WMD = 2.72 mg/dL, 95%CI = 0.47, 4.97; *p* = 0.02) (Figure 5(e)). The results of the sensitivity analysis showed that the heterogeneity may be derived from the Mafi et al. study [17]. The exclusion of the study reduced the heterogeneity to 55%, revealing that there was no significant reduction in the HDL-c (WMD = 1.56 mg/dL; *p* = 0.07). Subgroup analysis showed that there was a statistically significant difference in multiple species probiotic (WMD = 4.41 mg/dL, *p* = 0.009) and intervention duration > 8 weeks (WMD = 3.49 mg/dL, *p* = 0.01), which were better than the overall results (Table 2).

### 2.5. Effects of probiotics on inflammation and oxidative stress

#### 2.5.1. High-sensitivity C-reactive protein

**Figure 6.**
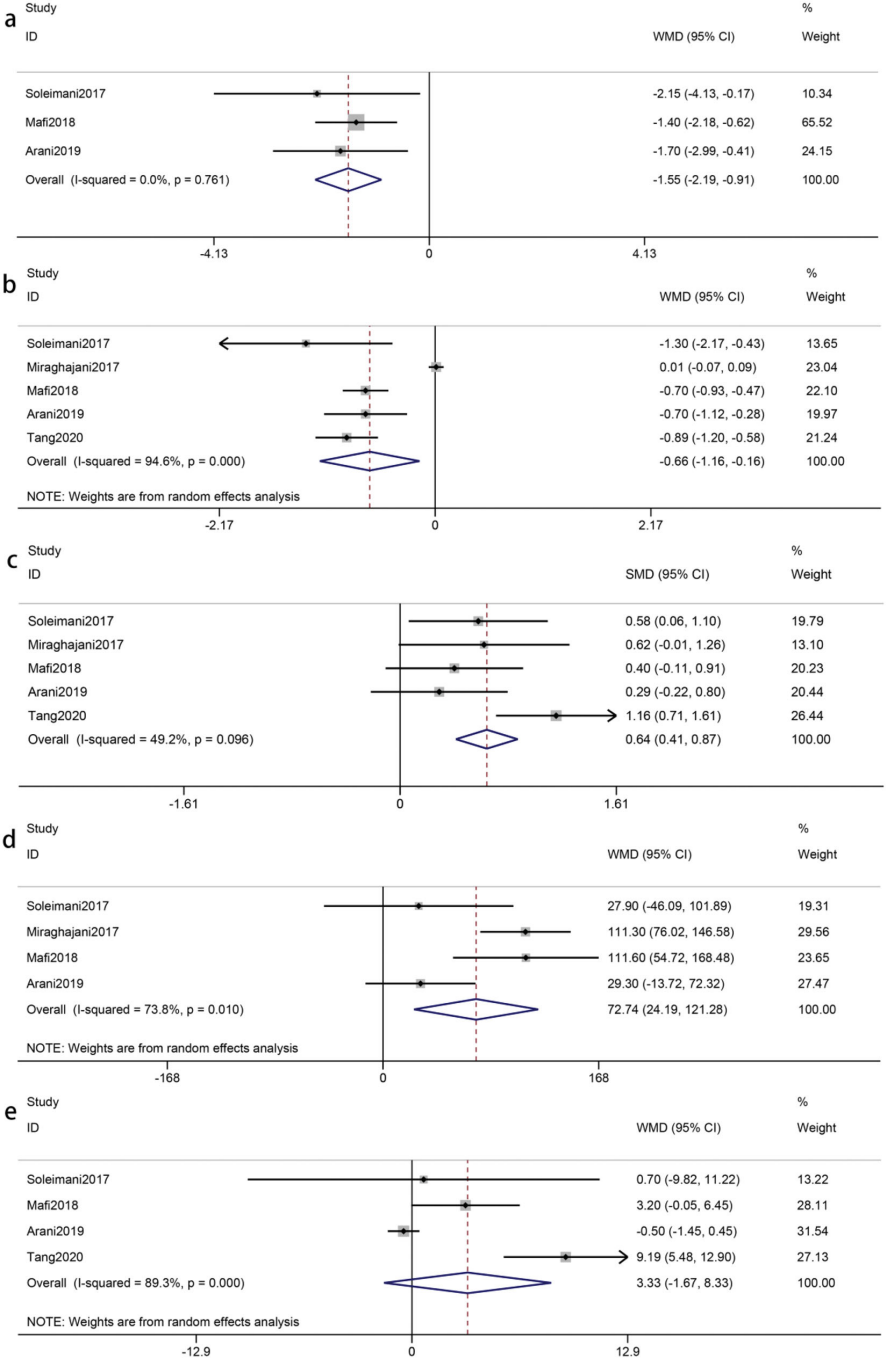
A. Effects of probiotics on inflammation and oxidative stress. (a) hs-CRP. (b) MDA. (c) TAC. (d) GSH. (e) NO. Abbreviations refer to Table 1.

The pooled results of three eligible studies [8,17,20] (intervention, 90; control, 90) revealed that probiotics decreased the hs-CRP of patients with DKD (WMD = −1.55 mg/L, 95%CI = −2.19, −0.91; *p* < 0.00001). Statistical heterogeneity was not identified (*I*^2^ = 0%, *p* = 0.76) (Figure 6(a)). Our study showed that the decreased hs-CRP was statistically significant in multiple species probiotics (WMD = −1.50 mg/L, *p* < 0.0001) (Table 2).

#### 2.5.2. Malondialdehyde

Five eligible studies [8,17,19,20,25] (intervention, 155; control, 155) showed that probiotics were associated with a reduced concentration of MDA in patients with DKD (WMD = −0.66 *u*mmol/L, 95%CI = −1.16, −0.16; *p* = 0.01). The test for heterogeneity was statistically significant (*I*^2^=95%, *p* < 0.00001) (Figure 6(b)). A sensitivity analysis found that the heterogeneity was derived from the Miraghajani et al. study [25]. The heterogeneity became an acceptable level after removing it (*I*^2^ = 0%, *p* = 0.48). The reduction in the MDA concentration as a result of probiotics was still significant (WMD = −0.77 *u*mmol/L; *p* < 0.00001). According to subgroup analysis, the impact of probiotics on MDA reduction was more apparent in intervention durations > 8 weeks (WMD = −0.77 *u*mmol/L, *p* < 0.00001), probiotic doses > 4*10^9^ CFU/day (WMD = −0.85 *u*mmol/L, *p* = 0.001) and multiple species probiotics (WMD = −0.80 *u*mmol/L, *p* < 0.00001) (Table 2).

#### 2.5.3. Total antioxidant capacity

Due to the different measurement units, the SMD was used. Five studies [8,17,19,20,25] (intervention, 155; control, 155) reported TAC, which was significantly different between groups (SMD = 0.64, 95%CI = 0.41, 0.87; *p* < 0.00001). There was no significant heterogeneity among the included studies (*I*^2^ = 49%, *p* = 0.10) (Figure 6(c)). Subgroup analysis showed that the impact of probiotics on TAC toward the subsets of ‘intervention duration > 8 weeks’ (SMD = 0.64, *p* < 0.00001) and multiple species probiotics (SMD = 0.75, *p* < 0.00001) was no less than the overall results (Table 2).

#### 2.5.4. Glutathione

The pooled of four studies [8,17,20,25] (intervention, 110; control, 110) showed a significant difference in GSH in the two groups (WMD = 72.74 mmol/L, 95%CI = 24.19, 121.18; *p* = 0.003). Meta-analysis of GSH indicated statistical heterogeneity was found (*I*^2^ = 74%, *p* = 0.01) (Figure 6(d)). Due to the heterogeneity, we conducted sensitivity analysis and subgroup analysis to identify possible sources. However, that could not explain the between-study heterogeneity (Table 2).

#### 2.5.5. Nitric oxide

Four studies [8,17,19,20] (intervention, 135; control, 135) for NO levels indicated that there was no significant difference in the probiotics group compared with the control group (WMD = 3.33 *u*mmol/L, 95%CI = −1.67, 8.33; *p* = 0.19). Significant heterogeneity was found in the meta-analysis (*I*^2^ = 89%, *p* < 0.00001) (Figure 6(e)). Sensitivity analysis showed that the overall results turned statistically significant (WMD = 5.26, 95%CI = 0.25, 10.27; *p* = 0.04) after the removal of the Arani *et al* study [20] with the heterogeneity (*I*^2^ = 69%, *p* = 0.04). Subgroup analysis found that there was a more apparent improvement when multiple species probiotics were used (WMD = 5.26 ummol/L, *p* = 0.04) (Table 2).

### 2.6. Publication bias

We examined publication bias by performing Egger’s test (quantitative). For improving inflammation and oxidative stress, our analysis revealed a significant difference in publication bias in hs-CRP and MDA (*p* = 0.021 and *p* = 0.033, respectively), but revealed no significant differences of publication bias in TAC, NO, and GSH (*p* > 0.05). For improving blood glucose, our analysis found that there was a publication bias in FBG and HbA1c (*p* = 0.004 and *p* = 0.004, respectively), but there was no evidence of publication bias for insulin, HOMA-IR, and QUICKI (*p* > 0.05). For improving renal function and blood lipids, our analysis showed no significant publication bias (*p* > 0.05) (Table 3).

**Table 3.** Egger’s test for publication bias.

Biomarkers	*N*	*I* ^2^	Egger’s test (*p* value)
Scr	6	0.004	0.372
BUN	5	0.001	0.403
GFR	5	0.05	0.155
Cys c	2	<0.00001	–
24 h-UP	2	0.36	–
UACR	2	0.004	–
K	3	0.39	0.837
Na	2	0.04	–
FPG	5	<0.0001	0.004
2 h PBG	2	0.09	–
HbA1c	4	0.002	0.004
Insulin	3	0.04	0.235
HOMA-IR	3	0.05	0.220
QUICKI	3	0.01	0.100
TG	4	0.1	0.744
TC	5	0.004	0.938
LDL-c	5	0.0003	0.790
VLDL-c	3	0.26	0.312
HDL-c	5	0.02	0.321
hs-CRP	3	<0.00001	0.021
MDA	5	0.01	0.033
TAC	5	<0.00001	0.405
NO	4	0.19	0.303
GSH	4	0.003	0.614

Abbreviations refer to Table 1.

## 3. Discussion

Regarding the beneficial effects of probiotics and the relationship between the imbalance of gut bacteria and the development of DKD, several studies have showed that probiotics are recommended for patients with DKD. The bacterial diversity, relatively distinct bacterial taxa at different levels, bacterially derived metabolites, and gut permeability in patients with DKD compared with healthy individuals, which were underlying mechanisms involving a vicious cycle of gut dysbiosis and DKD [26]. This meta-analysis, including 10 RCTs with a total of 552 participants, showed that compared with the control, probiotics in patients with DKD significantly ameliorated their renal function injury, improved biomarkers of glucose homeostasis and lipid metabolism, and benefited the improvement of inflammation and oxidative stress. In addition, our subgroup analysis revealed that probiotic intake patterns, probiotic dose, and duration of intervention affected the biomarkers.

Our meta-analysis demonstrated that probiotics could markedly delay the increase in Scr, BUN, Cys-c, UACR and Na in patients with DKD but had no effect on GFR, 24 h-TP, and K. Subgroup analysis revealed that when the intervention duration was longer than 8 weeks or the probiotic dose was not more than four billion CFU/day, Scr had a more apparent decrease. Interestingly, improvement of BUN after probiotic treatment was shown when probiotic doses administered were more than four billion CFU/day and multiple species probiotics were used, which might be due to multi-strain probiotics having synergistic interactions among different strains or a higher concentration of live cultures [27]. The majority of patients with DKD have disturbances in gastrointestinal tract mucus and the intestinal ecosystem, and have higher levels of aerobic bacteria such as *Escherichia coli* and lower levels of anaerobic microorganisms such as *Lactobacillus* and *Bifidobacterium*. Aerobic bacteria contribute to the high production of urea and an increased in pH levels. *Lactobacillus* and *Bifidobacterium* could decrease the pH through fermentation of carbohydrates, which prevents the proliferation of aerobic bacteria in the gut and promotes a balanced microbiota in the gut [28–30]. Furthermore, the urease activity of special probiotic species, such as *Bacteroides*, might improve urea degradation [31]. Probiotics enhance renal functions by reducing inflammation and pro-inflammatory cytokines, alleviating glomerular, and renal tubulointerstitial damage [32]. Moreover, probiotics provide sufficient substrates for the intestinal flora and promote the nitrogen-based growth of saccharolytic bacteria, therefore decreasing production of uremic toxins and preventing the aggravation of renal function loss [33]. The review by Vlachou [9] demonstrated the positive impact of probiotics on DKD without any major adverse events. Meta-analysis of Firouzi [34] between probiotics and blood parameters of renal function found that probiotics had a significant decrease in BUN, particularly when the intervention duration was less than 12 weeks and multi-strain probiotics were received. There were three meta-analyses of the association between probiotics and DKD. Tarrahi [12] showed that probiotics could reduce Scr, but BUN and GFR were no statistically significant changes. AbdelQadir [11] found that Scr, BUN, GFR, K and Na were nonsignificant between probiotic and control treatment, while the effect on Scr became significant after resolving heterogeneity by excluding one study. Wang [35] found that probiotics resulted in significant change in GFR, Scr, and BUN. The reason for the contradictory results was related to different sample sizes. Compared to previous studies, the number of subjects in our meta-analysis obtained increased. However, the reason that low-dose probiotics were more beneficial for Scr remained unclear and warrants further investigation.

Our meta-analysis revealed that probiotics improved glucose metabolism by regulating FPG, HbA1c, HOMA-IR, and QUICKI. However, there was no significat difference in 2 h-PBG and insulin. Further subgroup analysis showed that when multiple species probiotics were used or the probiotic dose was more than four billion CFU/d, FPG and HOMA-IR were more apparently reduced. However, Surprisingly, a dosage of <4*10^9^ CFU/day led to a more apparent significant decrease in HbA1c levels. HbA1c reflects the average level of blood glucose control over the past 2–3 months. The short intervention duration may account for the benefit of low-doses of HbA1c. In addition, the small number of trials in each subgroup or differences in probiotic strains may also be responsible. The mechanism of the hypoglycemic effect was that probiotics could affect intestinal flora to insulinotropic polypeptides and glucagon-like peptide-l (GLP-1), while these peptides induce glucose uptake by muscle [36]. Improved parameters of glucose homeostasis by probiotics might be due to reduced cytokines and suppression of the nuclear factor κ-light chain enhancer of the activated B-cells pathway [37]. Moreover, probiotics interact with gut flora and consequently produce metabolites such as short-chain fatty acids (SCFA) and bile acids, which could improve glycemic control and insulin sensitivity. Probiotics also regulate the secretion of proinflammatory mediators such as tumor necrosis factor-α, interleukin-6, and intestinal GLP-1 to improve glucose metabolism [38–40]. Tarrahi [12] reported a significant reduction in FPG and HOMA-IR in patients with DKD, with no significance for HbA1c, insulin, and QUICKI. AbdelQadir [11] showed a significant reduction in insulin with no effect on HOMA-IR, which is contrary to our results. It was associated with high heterogeneity. Neither sensitivity analysis nor subgroup analysis could better address heterogeneity, so the results should be interpreted with cautious.

In our study, a reduction in DKD-deteriorating risk factors, including TG, TC and LDL-c were observed. Further subgroup analysis showed that consuming single species probiotics and taking the probiotic dosage of <4*10^9^ CFU/d resulted in a higher reduction in TC and LDL-c. The duration of intervention presented contradictory results for TC and LDL-c, with long-term benefiting LDL-c and short-term benefiting TC. Some studies have demonstrated that probiotics intake could inhibit the host absorption of dietary cholesterol and suppress the reabsorption of bile acid in the small intestine [41]. Probiotics may help break down food-derived indigestible carbohydrates and increase the production of SCFA [42]. The resultant SCFAs could contribute to decreasing the cholesterol concentrations, either by inhibiting hepatic cholesterol synthesis or redistributing cholesterol from plasma to the liver [42]. SCFA also could regulate hormones controlling energy production and consumption (e.g., leptin and ghrelin). In addition, SCFA could improve insulin sensitivity and lipid profile by stimulating peptide YY and GLP-1 expression, which slows down digestive transit time, and by upregulating tight junction proteins and GLP-2, which decreases intestinal permeability [43,44]. Toll-like receptor 4 activation may explain the beneficial probiotic’s impact on the serum lipid profile [45]. AbdelQadir [11] found a marginally significant reduction in LDL but no benefits in TG, TC, LDL-c, and HDL-c. Moravejolahkami [10] indicated that lipid biomarkers such as TG, TC, LDL-c and HDL-c had a marginal reduction, but had no statistical significance (*p* > 0.05). The results of the above two meta-analyses were different from our results, perhaps due to the increase in our sample size and treatment heterogeneity. In addition, a meta-analysis [46] on T2DM patients showed that probiotics significantly decreased the serum levels of TC, TG and LDL-c compared with the control group, which gave support to our results. The effect of a single-species probiotic was better than a mixture of bacteria in TC and LDL-c. It may be that these different species inhibited each other, possibly by the production of antagonistic agents, or by competition for either nutrients or binding sites within the gastrointestinal tract [47]. Regarding the effect of different doses and intervention durations on the lipid profile, there was no clear mechanism, which needs further study.

Our meta-analysis revealed that probiotics significantly improved the levels of hs-CRP, MDA, TAC, GSH and NO in patients with DKD. Subgroup analysis suggested that among those used multi-strain probiotics, took high probiotics dosages or received the long-term intervention, there was a more apparent improvement in MDA, TAC and NO. Different probiotic bacterial strains could exert antioxidant effects with several suggested mechanisms including the following: (1) Probiotics can increase the production of SCFA in the gut and SCFA may block the enzymatic synthesis of hepatic CRP; (2) Probiotics can capture metal ions such as ferrous and cupric ions, which prevent metal ions from catalyzing oxidation processes; (3) Probiotics can make use of their antioxidant enzymatic systems such as superoxide dismutase and catalase; (4) Probiotics can produce various metabolites with antioxidant properties such as GSH, butyrate and folate; (5) Probiotics can protect against oxidative stress *via* regulation of the Nrf2-Keap1-ARE, mitogen-activated protein kinase, nuclear factor-κB and protein kinase C pathways; (6) Probiotics also can regulate the enzymes responsible for the production of ROS, which decreased the activity of the NADPH oxidase, cyclooxygenase and cytochrome P450 enzymes [48–50]. Similar to our findings, probiotics significantly reduced serum hs-CRP and MDA levels and increased oxidative parameters such as TAC, NO, and GSH in T2DM patients and subgroup analysis showed that an intervention duration of 12 weeks resulted in improvement by significantly increasing TAC levels and decreasing MDA levels [51]. Bohlouli [52] and Wang [35] showed that probiotics had a beneficial effect on inflammation and oxidative stress biomarkers by significantly reducing hs-CRP and MDA as well as increasing GSH and TAC, but there was no significant effect on NO. Subgroup analysis indicated that the overall effects of probiotics on serum TAC levels may be more pronounced at probiotic doses > 5 billion CFU/day. AbdelQadir [11] found that probiotics did not affect GSH, and NO but reduced hs-CRP, and MDA levels and increased the TAC levels.

Our meta-analysis has several limitations: Firstly, the number of RCTs included in the study was relatively small and studies included in this meta-analysis had follow-up periods ranging from 8 to 12 weeks, which were relatively short-term. Secondly, the patients included in this meta-analysis were different in ethnicity, eligibility criteria, disease progression, types and dosages of probiotics taken and follow-up times, which may have impacted our findings. Thirdly, the meta-analysis was not registered online. Finally, bias was inevitable. RCT, which reduces the bias to a certain extent, was analyzed in this meta-analysis, but the quality of the literature was reduced due to the differences in experimental design and measurement methods. The subgroup analysis also had some limitations. The limited number of included studies resulted in tiny subgroups, which weakened the generalizability of outcomes. Despite the above shortcomings, the reliability of this meta-analysis was strengthened by minimized incorporation of biased literature, rigorous data extraction, strong statistical analysis, stringent inclusion criteria and bias and sensitivity analysis. The results of this study are still worthy of clinical reference.

## 4. Conclusion

Our meta-analysis based on ten RCTs demonstrated that probiotics among patients with DKD had a beneficial effect on the metabolic indicators including renal function (Scr, BUN, Cys-c, UACR, Na), glucose homeostasis (FPG, HbA1c, HOMA-IR, QUICKI), lipid metabolism (TG, TC, LDL-c), inflammation and oxidative stress (hs-CRP, MDA, TAC, GSH, NO). Subgroup analysis revealed that a significant change was likewise observed for high-doses in BUN, FPG, HOMA-IR, MDA, multi-stain probiotics in BUN, FPG, HOMA-IR, HDL-c, MDA, TAC, NO, and long- term in Scr, LDL-c, HDL-c, MDA, TAC. However, a larger sample size, polycentric, and long-term follow-up RCTs will be necessary for the future to further clarify the therapeutic effects of probiotics on patients with DKD. Probiotics could become an effective and low-cost treatment for patients with DKD.
